# First Aid Practices and Health-Seeking Behaviors of Caregivers for Unintentional Childhood Injuries in Ujjain, India: A Community-Based Cross-Sectional Study

**DOI:** 10.3390/children5090124

**Published:** 2018-09-06

**Authors:** Ashish Pathak, Nitin Agrawal, Love Mehra, Aditya Mathur, Vishal Diwan

**Affiliations:** 1 Department of Pediatrics, R. D. Gardi Medical College, Ujjain 456006, India; lovemehra2000@gmail.com (L.M.); dr.adityamathur121@gmail.com (A.M.); 2 Department of Women and Children’s Health, International Maternal and Child Health Unit, Uppsala University, SE-751 85 Uppsala, Sweden; 3 Global Health—Health Systems and Policy, Department of Public Health Sciences, Karolinska Institutet, SE-171 76 Stockholm, Sweden; vishaldiwan@hotmail.com; 4 International Centre for Health Research, Ujjain Charitable Trust Hospital and Research Centre, Ujjain 456006, India; 5 Department of Paediatric Surgery, R. D. Gardi Medical College, Ujjain 456006, India; drnitinagrawal73@gmail.com; 6 Department of Public Health & Environment, R. D. Gardi Medical College, Ujjain 456006, India

**Keywords:** unintentional childhood injuries, first aid, health seeking, community survey injuries, India

## Abstract

Data on types of community first aid use and treatment provided post-injury from many low‒middle-income countries, including India, are lacking. This cross-sectional study was conducted among children aged one month to 18 years of age, in Ujjain, India, to understand types of first aid given and health-seeking post-injury. A total of 1087 injuries in 1049 children were identified in the past year. A total of 729 (67%) injured children received first aid and 758 (70%) sought some form of health care. Children with burns received the most (86%) first aid, and most children (84%) with road traffic accidents (RTA) sought health care. Most children (52%) sought health care from a private health care facility; most children (65%) were transported to a health care facility within the golden hour. Motorbikes were the most preferred (50%) mode of transport. Only 1% of the injured used ambulance services. Commonly reported methods or substances for first aid included the use of coconut oil on wounds from falls (38%) and burns (44%), the use of antiseptic cream on wounds from RTA (31%), the application of turmeric for wounds from falls (16%), and rubbing of metal on a bitten area (47%). For most injuries, appropriate, locally available substances were used. Potentially harmful substances applied included lime, toothpaste, clay, and mud. The findings will help design community interventions to increase the provision of appropriate first aid for childhood injuries.

## 1. Introduction

Unintentional childhood injuries are a leading cause of childhood mortality and morbidity [1,2]. According to the World Health Organization (WHO)’s Global Burden of Disease Study estimates, unintentional injuries accounted for 3.9 million deaths in 2004 [3]. In the young age group of 15–29 years, five of the 15 leading causes of death are related to unintentional injury [1]. Most injuries, particularly those among children from low‒middle-income countries (LMICs), lead to considerable morbidity and costs to individuals and society [4].

The burden of injury is highest in LMICs, where health systems’ priorities are skewed towards the treatment and prevention of communicable diseases, and injuries as a public health problem are often neglected [5,6]. In many LMICs, injuries are viewed as “accidents” that are outside of parents’ control [7]. Therefore, injuries are not analyzed, and no action is taken towards their prevention [7]. Timely and appropriate treatment of injuries can help reduce injury-related mortality and morbidity [8,9]. However, data on childhood injuries and response to injuries by caregivers of children from LMICs, particularly South Asia and India, are lacking [5,9,10,11,12,13,14,15,16,17,18]. Globally, studies evaluating first aid knowledge and practices are often conducted in patients with burn or trauma and are most often hospital-based [19,20,21,22,23,24,25]. A community-based study comprehensively investigating first aid practices and health-seeking behaviors for all injuries is necessary, because hospital-based studies are affected by the health-seeking pattern of the community and the availability of health care services. The present study primarily aimed to understand first aid practices and health-seeking behavior for common childhood injuries such as falls, road traffic injuries, burns, nonfatal drowning, poisoning, and suffocation. Since educational interventions can improve parental knowledge on safety both at home and outside home [7,26], the secondary objective of the study was to identify gaps in caregivers’ practices and health-seeking behaviors to design effective, context-specific interventions.

## 2. Materials and Methods

### 2.1. Study Site, Study Population, and Sampling

This descriptive cross-sectional study was conducted from January 2017 to October 2017. The details of study area, study population, sampling and sample size calculation are provided in a previous study [27]. In brief, the study was conducted by the Department of Pediatrics, R.D. Gardi Medical College (RDGMC), in both urban and rural areas of Ujjain, a semi-rural district in western Madhya Pradesh, India. Ujjain district has a population of 1.9 million within an area of 6091 km^2^ [28]. In urban and rural areas of Ujjain, 26% and 30% of the population are aged below 15 years, respectively [29]. As the rural area, seven villages were randomly selected from the Demographic Surveillance Site (DSS) of RDGMC [30]. As the urban area, 10 geographically contiguous slums in Ujjain City, having 2000 households with 10,000 individuals, around the Urban Health Center of RDGMC were selected. According to the WHO guidelines for sample size calculation for community survey of injuries, the minimum sample size was calculated to be 1173 children each for the rural and urban areas [31]. No compensation was provided to the study participants. The survey included 2907 and 3401 children from rural and urban areas, respectively, with a response rate of 98% [27].

### 2.2. Data Collection Tools and Methods

All the households in the sampling frame were visited, and households having children aged one month to 18 years of age were approached to participate in the study. Signed informed consent was obtained after the potential study participants were provided with an explanation of the study purpose. Three trained study assistants interviewed the female head of the household, along with two team leaders who supervised the data collection. A semi-structured questionnaire was used to interview caregivers to understand their first aid practices and health care-seeking behavior.

The questionnaire was originally developed in English and then translated into Hindi by two experts in the Hindi language [32]. Any discrepancy in the translated version was resolved through consensus by an expert panel, which consisted of two pediatricians not involved in the study [32]. The questionnaire was then translated back into English to ensure that the original meaning of the questions had not changed. The questionnaire was used to collect information on whether first aid was provided at the time of injury, the details of the person providing the first aid, and the details of the first aid applied. The questions related to health-seeking behaviors collected the details of the type of health-seeking settings, the mode of transport used, transport time and whether the child was hospitalized or not for the injury. The applicability, context and face validity of the questions was determined in a pilot test of 50 randomly selected caregivers [32]. The results of the pilot were not included in the final analysis. To collect missing data, if any, a revisit to the household was made within one month of the first visit, and if no one was found at home when the subsequent visits were made the household was considered to be a nonresponse [33].

### 2.3. Definitions

For the survey, the WHO definition of injuries was used; that is, injuries are caused by acute exposure to physical agents such as mechanical energy, heat, electricity, chemicals, and ionizing radiation interacting with the body in amounts or at rates that exceed the threshold of human tolerance [33]. The study assistants briefly explained the WHO definition of an injury and provided examples of external causes of injuries [33]. Any injury, as defined above, in the preceding 12 months of the survey was included in the analysis. Data on road traffic injuries, falls, burns, nonfatal drowning, poisoning and bites, and suffocation were collected and published in detail elsewhere [27]. Moreover, in this study, first aid was defined as emergency care or treatment provided before regular medical aid could be obtained. Health care-seeking behavior was defined as any care sought outside of the home for a child who sustained an injury [13].

### 2.4. Data Management and Analysis

Field data were collected through paper-based questionnaires. All paper-based questionnaires were reviewed daily for consistency and completion by the principal investigator and co-investigators. The data were coded and entered in EPI INFO (version 7). Analysis was performed using STATA 13 (StataCorp., College Station, TX, USA). The data were analyzed to determine the frequency of first aid practices and health care-seeking behaviors of caregivers. Chi square and Fisher’s exact tests were used to assess the difference between children who received first aid and those who did not. A *p* value < 0.05 was considered statistically significant. The crude odds ratio (OR) and corresponding 95% confidence intervals (CI) and *p* values were calculated from 2-by-2 tables. The study was approved by the Institutional Ethics Committee of RD Gardi Medical College, Ujjain (Approval No.-354/2014).

## 3. Results

In the survey, 2846 households were visited, of which 2518 were identified as having 6308 children aged up to 18 years. A total of 2907 and 3401 children lived in 1304 urban and 1214 rural households, respectively. The overall response rate was 98%. The remaining 2% percent of households could not be included in the study as they were locked even after follow-up visits. Of the 6308 children, 1049 children with a total of 1087 unintentional injuries were identified in the survey. A total of 729 (67%) injured children received first aid, 758 (70%) sought some form of health care, and 346 (32%) both received first aid at the injury site and sought health care post-injury.

Table 1 shows the age, sex, and urban and rural location of 1049 children with unintentional injuries and the proportion of children that received first aid. Significantly more girls received first aid than boys (OR 1.48, 95% CI 1.216–1.952; *p* = 0.005). No statistically significant differences were observed in the proportions of children receiving first aid according to age and urban or rural location of the households.

Of 1087 injuries, 729 (67%) received some form of first aid. Table 2 presents the proportion of injuries that received first aid according to different injuries. Most (86%) burn injuries received some form of first aid. However, only eight out of 25 (32%) non-fatal drowning injuries received some form of first aid.

**Figure 1:**
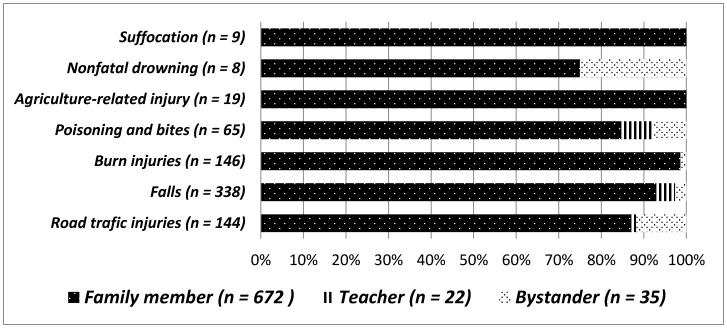
First aid care providers in different injuries.

For most injuries, first aid was commonly provided by the family members: in some cases, first aid was provided by teaches and bystanders. Figure 1 provides details of first aid care providers according to different injuries.

Information on the type of health care facility was available for 758 (70%) of injuries. Children with road traffic injuries, poisoning, or bites were most often taken to health care facilities post-injury (Table 3). Overall, parents of more than half of injured children went to a private setting for health care post-injury. Many children with falls (30%) were taken to an informal health care provider (Table 3). In rural areas, untrained informal health care providers had more of a presence than trained doctors and were the preferred option when seeking health care. 

The mode of transport for 758 injured children is shown in Table 4. Most children (47%) were transported by motorbike from the site of injury to the health care facility. Children living in rural areas were transported by public transport even if they had burns or poisonous bites. Twenty percent of children walked to a health care facility or private health care provider to seek health care post-injury. Ambulance services were used for only 1% of injures.

**Figure 2:**
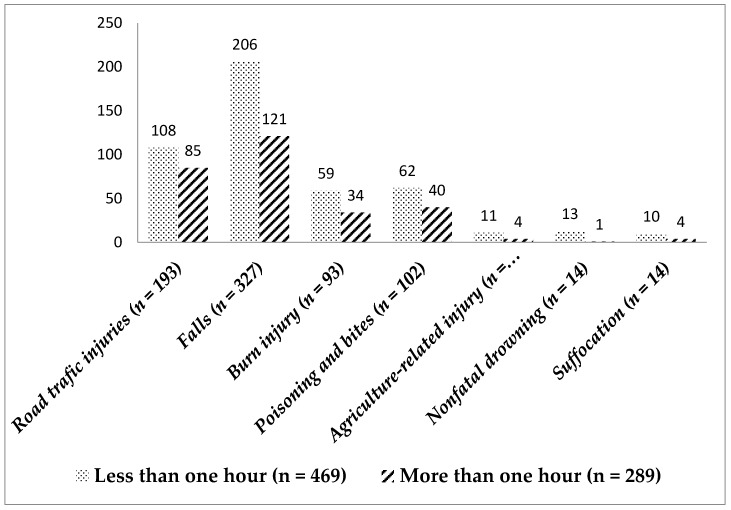
Health care-seeking with the golden hour in different injuries.

The majority (65%) of children were transported to a health care facility within 1 h, which is the golden hour after injury. The details of health care-seeking within the golden hour for different injuries are shown in Figure 2.

The details of first aid provided in different types of injuries are shown in Table 5.

Other substances applied over wounds and not shown in Table 5 were as follows: aloe vera (5%), mud (3%), toothpaste (4%), saliva (4%), and one’s own urine (3%). As first aid, butter (6%) and ice (5%) were used to treat burn injuries. For poisoning injuries caused by animal bites, tourniquet use was reported in a few children (4%); however, spiritual activities (3%) were reported for snakebites (*n* = 20).

## 4. Discussion

To the best of our knowledge, this is the first community-based survey to identify the type of first aid provided and health-seeking behavior following unintentional childhood injuries in Central India. In many LMICs, including India, the emergency medical health care system is fragmented and inaccessible [34]. Moreover, in India, there is poor awareness regarding the importance of correct pre-hospital care [34]. This study is an extension of a previous study, which determined the prevalence of unintentional injuries in the study area [27]. First aid is the care provided at the sight of injury (at home, school, work, or recreation area) or even during transportation until the patient arrives at a formal health care facility. However, few other studies have reported the proportion of injured children who received first aid. A study conducted in Bangladesh reported that 82% of injured children received some form of first aid [9]. In our study, most children with burns (86%) received some form of first aid. This proportion is comparable to those reported in studies conducted in Zimbabwe and Bangladesh [9,18]. In our study, the lowest proportion of children who received first aid was for nonfatal drowning and suffocation injuries (Table 2). The possible reason for a lower proportion of drowning and suffocation injuries receiving first aid is that these injuries are considered medical emergencies and neither family members nor bystanders are typically skilled enough to provide first aid treatment for such injuries.

First aid providers play a crucial role in the timely management of injuries. In our study, first aid providers were mainly family members. Few studies have identified first aid providers for injuries. However, a systematic review of first aid providers of trauma victims showed that bystanders provided first aid in 11–65% of the situations in various studies [35].

In our study, most injured children were taken to private health care providers in both urban and rural areas. In rural areas, they were taken to untrained or informal health care providers. The informal health care providers are defined as a diverse set of health care providers who have not received any formal education, who collect payments from patients served and not from institutions, and who are not registered with any government regulatory bodies [36]. A study done in Ujjain district found that untrained or informal health care providers constituted 56% (*n* = 1162) of the total private practitioners (*n* = 2075) in the study region [37]. The reported reasons for preference of informal health care providers are close proximity, availability, flexible opening times, options for payment-in-kind, perceived accountability, trustworthiness and, most importantly, affordability [36]. Informal health care providers are active in India, Bangladesh, Nepal, Laos, Kenya, Nigeria, and Tanzania; however, very few studies have evaluated their services provided for treating injuries. A study conducted in rural Bangladesh investigated the health-seeking behavior of study participants with informal health care providers and reported findings similar to our study for health-seeking [9].

In our study, almost 50% of patients were transported using motorbikes. In emergency medical services (EMS), motorbikes are underutilized; they have been found to be useful in heavy traffic situations and in resource-constrained countries such as Taiwan [38]. In our study, ambulance services were used as the mode of transport to a health facility for only 1% of injured children. In India EMSs are fragmented. Recently, the Government of India and state governments have made efforts to make ambulance services more accessible, including “dial 108” services, which are available round the clock and free of cost [34]. However, most efforts have been concentrated in emergency obstetric services, the utilization rate of which varies from 9% to 21% in different states across India [39]. EMS services should be optimized for a more satisfactory prehospital emergency care system, particularly for road traffic accidents, which were the most common injury in our study [34]. Recommendations to improve EMS include the administration of EMS at a more local level, providing improved training opportunities, optimizing the role of private sector in prehospital care, and improving public awareness on how to avail oneself of ambulance services [34].

In our study, 35–45% of injured children were not transported to a health care facility within the golden hour. Transporting the injured to a health care facility with 1 h (commonly known as the golden hour) can ensure the effective management of unintentional injuries and improves the outcome of injuries, particularly penetrating trauma, traumatic brain injury, and hypotensive trauma [8]. In the present study, we could not determine the outcome of injuries according to transport time because of the study design. Hospital-based surveillance can provide a correlation between the type of first aid provided and the outcome of the injury.

In our study, for first aid of burn injuries, the recommended practice of 10–20 min irrigation of the burn area with water was performed in only 13% of burn victims. Similarly, the nonuse of standard recommended first aid in burns has been reported in multiple studies [6,10,13,19,22,23,40,41].

In the present study, common household substances were identified as alternative therapies for first aid for various injuries. Antiseptic cream was the most frequently used first aid for management of cuts, wounds, and abrasions resulting from road traffic accidents, falls, and burn injuries. In the majority of cases, coconut oil was used to treat cuts, wounds, and abrasions. Coconut oil is also commonly used for wound management in another South East Asian nation, Indonesia [42]. Coconut oil is an example of an ancient compound whose application in skin repair is currently supported by modern science [43]. Coconut oil originates from the *Cocus nucifera* tree from the Indian‒Indonesian region [43]. Globally, coconut oil is commonly used as a topical therapy for xerotic and inflammatory dermatoses associated with skin-barrier disruption. However, its use for wound care and burn care has not been reported from other parts of the world, except in India and Indonesia. In the present study, it was commonly used for treating multiple types of injuries and burns (Table 5). Reasons for its widespread use in the study area may be its accessibility and inexpensive nature. Coconut oil has antimicrobial activity because it contains monolaurin, a monoglyceride formed from lauric acid, which is a short-chain fatty acid with antibacterial activity against *Propionibacterium acnes*, *Staphylococcus aureus*, and *S. epidermidis*, and this compound also has skin-barrier repairing properties for treating various skin conditions [43]. Although clinical data demonstrating the effectiveness of coconut oil in treating burns are insufficient, an Indian study has proved the usefulness of coconut oil in treating burn s [44]. Thus, the use of coconut oil by the community for mild burns can be considered appropriate first aid.

In our study, another commonly used Indian herb, known in India as *haldi*, was also used as first aid for cuts, wounds, and abrasions. Turmeric (*Curcuma longa*) is a popular ancient Indian herb that has been used for centuries in herbal medicines. The main alkaloid of turmeric is curcumin (diferuloylmethane), which has significant anti-inflammatory, anti-oxidant, anti-carcinogenic, anti-mutagenic, anti-coagulant, and anti-infective effects [45]. Curcumin has also been shown to have significant wound healing properties, as it acts in various stages of the natural wound healing process to hasten healing [45].

In our study, some inappropriate substances were used for treating injuries, particularly toothpaste for burn injuries, oil massage for physical injuries due to road traffic accidents and falls, and the use of spiritual activities as first aid for animal bites and poisoning. Some of the methods used by the caregivers can cause harm by delaying health-seeking from appropriate health care providers. Moreover, some of the substances like toothpaste for burns can result in wound infection and can cause harm. In African countries such as Ghana, South Africa, and Nigeria, similar alternative therapies have been reported following burn injury [10,13,18]. Previous studies have reported that raw egg whites, butter, milk, cooking oil, potato slices, yoghurt, toothpaste, tomato paste, ice, papaya, chalk, and salt are substances applied as first aid in countries such as Ghana, South Africa, Nigeria, South Africa, Turkey, and the United Kingdom [10,13,18,19,23,41,46]. Many of these substances were not applied in our study, reflecting the need for context-specific studies to improve pre-hospital management of injuries.

In this study, in the majority of bites, the bitten area was rubbed with a metal piece as a first aid measure. This practice was more common in rural areas. However, in some cases spiritual activities were used as treatment. Snakes have a huge connection with Indian mythology [47]. Rather than considering snakebite as an emergency, snakes are worshiped in India, and snakebites are considered to be a result of past sins [47]. A study conducted in Sindh province of Pakistan reported that 75% of patients treated snakebites by themselves or sought advice from traditional healers [48]. Similarly, studies conducted in South Africa and Kenya have shown that 80% and 70% of snakebite victims, respectively, received traditional treatment [49,50]. A study conducted in Sri Lanka reported that one-fifth of the snakebite victims initially received traditional treatment [51]. Similar findings have been reported in Nepal [52].

### 4.1. Strengths and Limitations

At present, India does not have a national injury database; therefore, this community-based household survey gives much-needed information on childhood injury prevalence in India. The study results are generalizable to similar resource-poor settings. However, all household surveys, including the present study, have the potential for recall bias due to unreliable memory. Also, social desirability could have inflated the proportions of first aid provided and health-seeking in certain types of injuries. We did not measure health literacy in the community included in the study, which has the potential to influence first aid and health-seeking. 

### 4.2. Recommendations

More translational and implementation research is required to establish the extent of first aid that should be given by first aid providers, to establish the substances that can be used as first aid, to determine how accurately the recommended guidelines are applied, and to establish specific measures that are required to improve emergency medical care in prehospital settings. Context-specific interventions must be identified and implemented. One example is to introduce and scale up lay training in first aid for injuries and basic life support (BLS) for improving prehospital services. The Indian Academy of Pediatrics’ BLS initiative is one such step, and should be scaled up [53].

## 5. Conclusions

In this study, for most injuries, the community used appropriate, locally available substances like coconut oil, antiseptic creams, and turmeric powder. However, some potentially harmful substances such as toothpaste, lime, clay, and mud were also applied over wounds. Most injured children were taken to a private health care provider post-injury. Low utilization of government health care facilities and ambulances for transport post-injury is a cause for concern. The reliance of the rural population on unqualified informal health care providers is also a cause for concern. Our findings will help design community interventions to increase the provision of appropriate first aid in childhood injuries.

## Figures and Tables

**Table 1:** Distribution of age, sex and urban and rural location of 1049 children identified with unintentional injuries and those who received first aid.

Variable	Children Having an Unintentional Injury *n* = 1049 (17*%)	Not Received First Aid *n* = 320 (31%)	Received First Aid *n* = 729 (69%)	OR	95% CI	*p* Value
**Sex**						
Boys	658 (20)	219 (33)	439 (67)	1.48	1.216–1.952	0.005
Girls	391 (13)	101 (26)	290 (73)			
**Age group**						
1 month–1 year	36 (8)	8 (22)	28 (78)	R	R	R
>1–5 years	276 (19)	95 (34)	181 (66)	0.73	0.322–1.691	0.473
>5–10 years	319 (19)	93 (29)	226 (71)	0.59	0.263–1.360	0.221
>10–18 years	418 (15)	124 (30)	294 (70)	0.51	0.229–1.160	0.110
**Location**						
Rural	540 (16)	154 (28)	386 (72)	0.92	0.713–1.198	0.553
Urban	509 (18)	166 (33)	343 (67)			

*% Row percentage, OR—Odds Ratios, CI—Confidence intervals.

**Table 2:** The proportion of injuries (*n* = 1087) receiving first aid according to the injury type and rural and urban distribution of 1049 injured children.

Injury Type	Total *n* = 1087	First Aid Given *n* = 729 (#%)	Rural Population *n* = 374 (51*%)	Urban Population *n* = 355 (49*%)
Road traffic accidents	229	144 (63)	73 (51)	71 (49)
Falls	491	338 (69)	168 (50)	170 (50)
Burns	170	146 (86)	58 (40)	88 (60)
Poisoning and bites	126	65 (52)	53 (82)	12 (18)
Agriculture-related injury	25	19 (76)	18 (95)	1 (5)
Nonfatal drowning	25	8 (32)	1 (13)	7 (87)
Suffocation	21	9 (43)	3 (33)	6 (66)

#% Column percentage, *% Row percentage.

**Table 3:** Distribution of health seeking and the place of health seeking for 758 injuries.

Type of Injury	Total Injuries *n* = 1087	Health Care		Place of Seeking Health Care (*n* = 758)		
		Not Sought *n* = 329	Sought *n* = 758 (#%)	Private Setting *n* = 393 (52*%)	Government Setting *n* = 194 (26*%)	Informal Health Care Providers *n* = 171 (22*%)
Road traffic injuries	229 (21)	36 (16)	193 (84)	103 (53)	55 (28)	35 (18)
Falls	491 (47)	164 (33)	327 (67)	160 (49)	69 (21)	98 (30)
Burns	170 (16)	77 (45)	93 (55)	53 (57)	23 (25)	17 (18)
Poisoning and bites	126 (12)	24 (19)	102 (81)	54 (53)	32 (31)	16 (16)
Agriculture-related injury	25 (2)	10 (40)	15 (60)	7 (46)	6 (40)	2 (13)
Nonfatal drowning	25 (2)	11 (44)	14 (56)	7 (50)	6 (43)	1 (7)
Suffocation	21 (2)	7 (33)	14 (67)	9 (64)	3 (21)	2 (14)

#% Column percentage, *% Row percentage.

**Table 4:** Mode of transport for injured children (*n* = 758) in different injuries.

Type of Injury	Total *n* = 758 (#%)	Two Wheeler @ *n* = 360 (47) *%	Public Transport *n* = 212 (28) *%	Walking *n* = 144 (19) *%	Others *n* = 36 (5) *%	Ambulance *n* = 6 (1) *%
Road traffic injuries	193 (25)	120 (62)	40(21)	24(12)	5(3)	4(2)
Falls	327 (43)	202 (62)	46 (14)	71(22)	7(2)	1(0)
Burns	93 (12)	14 (15)	44(47)	22(24)	12(13)	1(1)
Poisoning and bites	102 (13)	18 (18)	56(55)	19 (19)	9(9)	0
Agriculture-related injury	15 (2)	0	13(86)	1(7)	1(7)	0
Non-fatal drowning	14 (2)	2 (14)	5(36)	6(43)	1(7)	0
Suffocation	14 (2)	4 (29)	8(57)	1(7)	1(7)	0

*% Row percentage, #% Column percentage, @ Most commonly motorbike.

**Table 5:** Details of first aid provided in different injuries.

First Aid Used in Different Injuries	*n*	*%
**Road traffic injury (*n* = 144)**		
Antiseptic cream	45	31
Coconut oil	37	26
Bandage	31	22
Turmeric powder	22	15
Lime	14	10
Turmeric powder and quick lime	14	10
**Falls (*n* = 338)**		
Coconut oil	130	38
Antiseptic cream	119	35
Turmeric powder	64	19
Oil massage	32	9
Lime	38	11
Bandage	48	14
Turmeric powder and coconut oil	30	9
Turmeric powder and quick lime	38	11
**Burns (*n* = 146)**		
Coconut oil	64	44
Antiseptic cream	36	25
Toothpaste	32	22
Irrigation with water for 10–20 min over burn area	19	13
**Poisoning due to ingestion/inhalation (*n* = 16)**		
Washed with water and soap	9	56
Washed with water	6	38
**Poisoning and bites (*n* = 49)**		
Rubbed with metal on bitten area	15	31
Turmeric powder	10	20
Spiritual activities	22	45
**Agriculture injury (*n* = 19)**		
Coconut oil	6	32
Tourniquet	5	26
Bandage	4	21
Antiseptic cream	3	16
Washed with water	2	11
**Non-fatal drowning (*n* = 8)**		
Prone position	4	50
Mouth to mouth breathing	3	37
Pressed chest to remove water	1	13
**Suffocation (*n* = 9)**		
Hilted back to remove the airway obstruction	5	56
Used fingers to remove the airway obstruction	4	44

*% Row percentage; Only first aid types used in at least 10% of children in each injury type are shown in the table.
